# Influenza and Local Immunity

**Published:** 1906-12-22

**Authors:** Charles J. Whitby

**Affiliations:** Resident Physician Smedley's Memorial Hospital, Bath


### Hospital Clinics.

### INFLUENZA AND LOCAL IMMUNITY.

By CHARLES J. WHITBY, M.D.(Camb.), Resident Physician Smedley's Memorial Hospital, Bath.

Clinical observers must in recent years have been struck, as I myself have often been struck, with the fact that, though influenza is as common as ever, it is of a very different type from that with which we first made acquaintance some ten or twelve years ago. I refer to the great epidemic of so-called Russian influenza which, coming, as the name implies, from the East, overwhelmed us like a wave, and struck down whole cities within the space of a few hours. We read then of ships' crews rendered helpless, of factories and shops compelled to suspend activity, of households disorganised, and nurses at a premium. For influenza in those days was a veritable pestilence; the symptoms were acute, almost fulminating in type, and pyrexia or hyperexia was the rule. The Pfeffer bacillus had found in our over-civilised community a virgin soil, and rioted in an environment of pre-eminent predisposition. Yet, even in those early days, the variability of type so characteristic of this disease, was evident enough. The respiratory organs, the abdominal viscera, the brain or the peripheral nerves would be attacked, sometimes together, more often in rotation, and the disease by its protean manifestations almost mocked the diagnostic acumen of the profession. Year by year it reappeared in wave-like epidemics, always variable, yet with a gradual decline in the acuteness of individual manifestations. Now it has become endemic among us, and there are few adults who have not undergone several attacks. So far as my own practice goes, it has now become somewhat exceptional to meet with a case of typical acute influenza, with definite pyrexia, among adults past or near middle-age. Among infants it is much commoner; last winter, for example, there was in Bath a sharp epidemic of respiratory influenza, with broncho-pneumonia, among children less than two years old. Adults escaped infection for the most part, doubtless because the majority have by this time acquired a considerable degree of immunity by previous attacks. This is proved, I think, by the fact that those adult relatives who did not entirely escape were seldom ill enough to be confined to bed even for a day, whereas in the case of the infants, danger, and even death, was by no means rare.

Whether the influenzal epidemics recorded in history were of the same kind as the modern disease is extremely doubtful. For practical purposes what we now call by that name is, among Europeans, a new disease. An exceptional interest attaches, therefore, to the phenomena attending its establishment among us, and particularly to that extreme variability of type to which I have alluded above. There is a struggle for existence among diseases, and those which established themselves among us do so in proportion to a power of adaptability which is expressed in Darwinian phrase as the "survival of the fittest." What, from the clinical standpoint, might be called the evolution of influenza, from its primitive pyrexial to its present modified type among adults, throws, I think, some light on a question of interest in regard to the general theory of immunity. What is the reason that a person who has been the victim of numerous attacks of influenza seldom, if ever, displays the same symptoms in successive illnesses? If one year the attack has been attended by acute pharyngitis or tonsillitis, next year it may be ushered in by acute abdominal pain with or without diarrhœa and vomiting, possibly culminating in appendicitis or a mild form of general peritonitis. Or, again, there may be headache of agonising severity, suggestive of meningeal inflammation as the pathological basis. Recently, a tendency to ophthalmic complications, conjunctivitis, blepharitis, even glaucoma, has been forced upon my recognition. Surely this extreme variability, with an unvarying substratum of general toxæmic symptoms too characteristic to be easily overlooked, is in a high degree suggestive of a gradual exhaustion of the susceptibility of particular tissues and organs—the development of *local immunities* which narrow down the field of action of the bacillus and its toxines.

It is at present an open question whether the antitoxines, of admittedly cellular origin, which render the attacking bacillus of a given disease liable to engulfment by the defending leucocytes, are themselves of leucocytic or of tissue-cell origin. To me it seems that the observations generalised above distinctly favour the view that all, or at any rate, most tissue cells may have the power of defending themselves by elaboration of their own antitoxines. Such a power once developed by the protoplasmic elements of a given tissue or organ would not be easily lost, and would, on reinfection of the individual in question, at once reassert itself in defence of that tissue or organ. For we know that immunity, once established, has a decided tendency to persist; and immunity is practically synonymous with the power of elaborating specific antitoxines. And I submit that the theory of a local immunity dependent on the activity of the cell-elements of particular tissues, organs and regions of the body, explains the clinical characteristics of influenza in a way that is highly suggestive of its truth.

Finally, I would suggest for the manifestations of influenzal toxæmia in an individual protected by several acute attacks, for that modified form of the disease which is now so common, so elusive in a diagnostic sense, yet so obtrusive in the disabilities and sufferings of which its numerous victims are conscious—the name of the "*influenzal state*." I myself when in attendance upon acute influenza, though proof to a considerable extent against infection in virtue of previous attacks, am usually conscious of a distinctly toxæmic state. Headache in the morning, "rough throat," mild conjunctivitis, anorexia, undue sense of fatigue, meteoric abdominal pains, insomnia, neuralgias of various kinds—these, among others, are common symptoms of the "influenzal state," as I have experienced and observed it among the partially immune. Its existence is one more instance of the striking clinical analogy between the characteristics of malaria and influenza.

